# Does Quality of Life Act as a Protective Factor against Believing Health Rumors? Evidence from a National Cross-Sectional Survey in China

**DOI:** 10.3390/ijerph18094669

**Published:** 2021-04-27

**Authors:** Haixia Wang, Xiqian Zou, Kaisheng Lai, Weiping Luo, Lingnan He

**Affiliations:** 1 School of Journalism and Communication, Jinan University, Guangzhou 510632, China; whxpku@jnu.edu.cn (H.W.); zouxiqian@stu2019.jnu.edu.cn (X.Z.); kaishenglai@126.com (K.L.); weipingluo597@stu2020.jnu.edu.cn (W.L.); 2 School of Communication and Design, Sun Yat-sen University, Guangzhou 510006, China; 3 Department of Psychology, Sun Yat-sen University, Guangzhou 510006, China; 4 Guangdong Key Laboratory for Big Data Analysis and Simulation of Public Opinion, Guangzhou 510006, China

**Keywords:** quality of life, WHOQOL-BREF, health rumor belief, social media

## Abstract

A high quality of life (QoL), an individual’s subjective assessment of overall life condition, has been shown to have a protective effect against negative behaviors. However, whether QoL protects people from the harmful impact of health rumors is still unknown. In this study, a national survey in China (n = 3633) was conducted to explore the relationship between health rumor belief (HRB) and QoL, which includes physical, psychological, social, and environmental domains. The results show that people with a poor perception of their physical health are more likely to believe health rumors. Additionally, those who had better self-reported satisfaction in social relationships were more susceptible to health rumors. Furthermore, women and older adults showed a greater belief in health rumors. This study expands upon our understanding of how people with different QoL levels interact with false health-related information. Based on health-rumor-susceptible groups, several essential online and offline strategies to govern health rumors are also proposed.

## 1. Introduction

Quality of life (QoL), an indicator of multidimensional aspects of personal living conditions, has been proved to be associated with happiness, well-being, and life satisfaction [1]. Maintaining and developing QoL for human beings has become the main criterion for sustainable development [2] and the center of social policy [3]. In the field of health care, QoL is often considered a critical health outcome [4]. Thus, studies have been conducted to identify vital factors of QoL and to improve the QoL of individuals. For instance, the results of a global cross-cultural survey indicated that daily living activities, having energy, and overall health are the three most important factors influencing QoL [5].

It has been argued that higher QoL plays a positive role in promoting personal development. For instance, higher QoL was accompanied by better academic performance [6], better interpersonal trust [7], and was considered a robust predictor of longevity [8]. By contrast, lower QoL is associated with several adverse emotional states and behaviors, such as self-sustaining stress [9,10], body dissatisfaction [11], and problematic Internet use [12]. Moreover, according to a cross-sectional survey, individuals with a higher score in the psychological and social domains of QoL showed lower alcohol dependence [13]. Based on the aforementioned studies, higher QoL serves as a protective mechanism for certain negative behaviors to some extent. However, whether the protective mechanism of QoL also works in keeping people from believing health rumors is still unknown.

### Literature Review

Health rumors, as risky information statements, with unverified facts, may lead individuals or groups to take inappropriate or irrational actions related to their health. Moreover, health rumors are widespread on the Internet [14,15]. According to a rumor governance report released by Tencent, one of the largest Internet companies in China, in 2018, about 1.4 million online rumors have been identified per day, of which health rumors were considered to occupy the largest proportion [16]. Especially in a public health crisis, the negative impact of health rumors becomes more salient. For example, during the outbreak of the COVID-19 epidemic, a health rumor claiming that “Dual-yellow Oral Liquid” could treat COVID-19 caused people to panic-buy the herbal medicine, which impacted the political and economic order of the society. Considering their unverified nature, the prevalence of health rumors may cause serious consequences [17], such as inappropriate health-detection and disease-prevention behaviors [18]. In response to the increasing proliferation of health rumors on the Internet, extensive efforts have been made to govern health rumors. However, the first step of health rumor governance is to understand why people believe them [19], which is the primary basis for preventing their further dissemination. To explore the underlying mechanism behind rumor belief, previous studies have examined several external factors that affect rumor belief, such as the content characteristics of rumors [14,17,20], crisis [21], and platform characteristics of media synchronicity [22]. Conversely, numerous efforts have targeted rumor-susceptible people because their characteristics, for example, personality traits [19], personal involvement [23], psychological stress and anxiety state [24,25], and existing attitudes [26] can also predict a significant relationship with rumor belief. As a multifaceted concept, QoL has been regarded as an indicator of an individual’s living conditions [1]. Thus, QoL could also be an aspect of an individual’s characteristics. However, whether QoL can act as a protective factor against believing health rumors is still unexplored. In addition, health rumors could cause serious consequences. For example, during the 2011 Fukushima disaster in Japan, an online rumor claiming that eating salt could prevent radiation damage caused panic-buying of salt in China’s coastal areas [27]. There is value, then, in examining whether QoL protects people from such health rumors. This study aimed to investigate the relationship between QoL and health rumor belief (HRB).

Considering that high QoL has a protective effect on health risk factors, it was speculated that it may also play a role in protecting people from believing health rumors. Previous studies have found that individuals with higher psychological anxiety are more likely to believe health rumors [25,26], while higher QoL was associated with lower psychological anxiety [28]. Thus, a high QoL might be associated with resisting HRB via low psychological anxiety. Accordingly, this study aims to explore the relationships between HRB and the four classification domains of QoL by identifying which kind of people are more likely to believe in health rumors. The hypotheses of this study were as follows:

**Hypothesis 1** **(H1).** *Higher QoL in the physiology domain will be negatively associated with HRB.*

**Hypothesis 2** **(H2).** *Higher QoL in the psychology domain will be negatively associated with HRB.*

**Hypothesis 3** **(H3).** *Higher QoL in the social relationship domain will be negatively associated with HRB.*

**Hypothesis 4** **(H4).** *Higher QoL in the environmental domain will be negatively associated with HRB.*

## 2. Materials and Methods

### 2.1. Sample and Procedure

This study was conducted using an online questionnaire, which was promoted via the Tencent Questionnaire platform (https://wj.qq.com/ (accessed on 28 November 2018)), an online questionnaire collection platform with nearly 3 million active users per day [29]. The online questionnaire system requires participants to complete each question before starting the next one or submitting the questionnaire. Only the data of participants who completed all of the questions were collected, so there were no missing data. All participants were anonymous and volunteered to fill in the survey without any extrinsic incentive. All users’ IP addresses were recorded by the questionnaire system and, therefore, the survey could only be accessed once from each device. After participants finished the survey, they were informed that the information texts for judgement were identified as false rumors by a fact-checking website. Contact information was provided for participants to ask any questions they had about this research. In this survey, participants were asked to read four false health rumors identified by the Tencent Jiaozhen (fact-checking) website [30] and indicate their belief in each: (1) most of the “fast growing ducks” on the street with a growth period of one month contain hormones; (2) soybean milk, radish and Sydney have the effect of “clearing lung” and can fight against haze; (3) eating fruit on an empty stomach can cure cancer; (4) onion in the room can prevent flu. Then, participants completed the World Health Organization Quality of Life Instrument, Short Form (WHOQOL-BREF) scale to report their QoL level. Finally, participants answered additional demographic questions added to the questionnaire. The research was examined and approved by the Ethics Committee of the Department of Psychology, at Sun Yat-sen University (China).

### 2.2. WHOQOL-BREF

The WHOQOL-BREF scale was used to explore the relationship between HRB and QoL, including its four major domains (physical, psychological, social relations, and environmental). The standard QoL measurement and definition of the World Health Organization (WHO) are used in this study. According to the WHOQOL group, QoL refers to a broad concept that includes “persons’ physical health, psychological state, level of independence, social relationships and their relationship to salient features of their environment” [31]. As an interdisciplinary and multidimensional concept, QoL has been applied in many fields, such as environment, medicine, recreation, nursing, psychology, sociology, economics, and so on [32,33,34]. WHOQOL-BREF, as a simplified version of WHOQOL-100 proposed by WHO, has been widely used to assess QoL in many areas. The reliability and validity of the electronic version of the WHOQOL-BREF also have been verified [35].

The WHOQOL-BREF questionnaire, a short version of WHOQOL-100 [36], has been translated into more than 20 languages [37] and made available to Chinese researchers since 2000 [38]. The WHOQOL-BREF is classified into four major domains, namely, physical (7 items), psychological (6 items), social relationships (3 items), and environmental (8 items). Each item is rated on a five-point Likert-Scale, among which three negatively worded items are reversed scored [39,40], with higher scores indicating a better QoL [41]. As indicated in the user manual, each domain score is multiplied by 4 to be directly comparable with scores derived from the WHOQOL-100 [42]. The internal consistency of the instrument was measured by Cronbach’s α, which ranged from 0.67 to 0.82 (physical domain 0.79, psychological domain 0.82, social relationships domain 0.67, environmental domain 0.84).

### 2.3. Health Rumor Belief

The four health rumors were rated on a five-point scale (1 = extremely doubtful, 2 = doubtful, 3 = uncertain, 4 = believable, 5 = extremely believable) indicating the extent to which participants believed in each of them; higher scores indicated higher HRB toward the corresponding item. In this study, two different methods were adopted to measure HRB separately. First, FFHR (falling for health rumors) was obtained to reflect whether people believed in health rumors. More specifically, the values of “uncertain,” “believable,” or “extremely believable” (“3,” “4,” or “5” on the five-point scale) were identified as “Falling” for health rumors. The four rumors were verified as false; if people were uncertain whether they are rumors, they may be “Falling” for them. Conversely, “extremely doubtful” or “doubtful” (“1” or “2” on the five-point scale) meant a solid skeptical attitude toward the health rumor, which was identified as “Not-Falling” for health rumors. Among the four rumors, if the participants believed even one health rumor, the researcher assigned them an FFHR score of 1, and if they did not believe any rumor, they were assigned a score of 0. Second, to test the robustness of the results, HRB was treated as a dichotomous variable. Referring to previous studies assessing people’s general rumor belief [19], HRBS (health rumor belief score) was obtained by averaging the four health rumor items on the scale.

### 2.4. Demographic Variables

Demographic variables, such as gender, age, residence (urban area, rural area), education (less than middle school, technical college, Bachelor’s degree, Master’s degree or higher), and monthly household income (<2000 RMB, 2001–6000, 10,001–15,000, 10,001–15,000, 15,000–30,000, 30,001–45,000, 30,001–45,000, >60,000 RMB), were also included.

### 2.5. Data Analysis

Data analysis was performed using SPSS for Windows Version 22.0 (IBM Corp, Armonk, NY, USA). Descriptive statistics were used to analyze socio-demographic characteristics, HRB, and QoL, where all domain scores of QoL were converted in accordance with the guidelines of the WHOQOL-BREF user manual [42]. The linear regression analysis to test the relationship between individual QoL level and HRBS was used. Logistic regression was also used to test the relationship between HRB and QoL because FFHR is a dichotomous variable. A two-tailed *p* < 0.05 was considered statistically significant.

## 3. Results

The survey collected a sample of 3633, which varied in demographic characteristics, such as gender (55% males, 45% females), age (mean = 30.7 ± 9.4), and residence (25.3% rural area, 74.7% urban area) (Table 1).

Scores of the four major WHOQOL-BREF domains and HRB are shown in Table 2. Among the WHOQOL-BREF domains, the highest mean score was of the physical domain (14.96 ± 2.74) and the lowest mean was of the social domain (13.33 ± 3.37). The average score of HRBS was 2.89 (SD = 0.84), indicating that people believed health rumors to some extent. On a more stringent level, the average score of FFHR was 0.84 (SD = 0.37), suggesting that about 80% of people believed health rumors. The results show that the female group were more likely to believe in health rumors than the male group (t = −7.782, *p* < 0.0001); the older people (above 50) were more likely to believe in health rumors than people below 18 (mean difference = 0.394, *p* < 0.01) and people in the 18–29 age group (mean difference = 0.276, *p* = 0.05). Although the results were not significant, the pattern was consistent with that of the previous two groups: the older people (above 50) were more likely to believe in health rumors than people in the 30–39 (mean difference = 0.253, *p* = 0.105) and 40–49 (mean difference = 0.223, *p* = 0.296) age groups.

To explore the relationship between QoL and HRB, we replaced HRBS with FFHR and constructed a logistic regression model to test the relationship between the four domains of QoL and FFHR (Table 3). According to the results of the logistic regression model, there was a significant negative correlation between the physical domain of QoL (B = −0.060, *p* = 0.037) and FFHR. After controlling for relevant demographic variables, the result still supported H1. Although there is a marginally significant effect between FFHR and the social domain of QoL (B = 0.036, *p* = 0.088) in the logistic regression model, the positive relationship pattern between them still could be observed. As with the result pattern in HRBS, H2 and H4 were not supported in FFHR. What is more, gender (B = 0.477, *p* < 0.001) and age (B = 0.201, *p* = 0.001) were still significant predictors.

To further test the robustness of the relationship between HRB and QoL, we constructed a linear regression model including the four QoL domains to predict HRB. The variance inflation factors (VIFs) of the four domains of QoL are 2.447, 2.802, 2.207, and 2.494 when predicting the HRB, which are much smaller than 10. Based on these results, collinearity is not a significant problem. This is consistent with the previous studies that also use the four dimensions of QoL [43]. The predictors of HRBS among all participants are presented in Table 4. According to the results of the linear regression analysis (Model 3), there was a significant relationship between the HRBS and physical domain of QoL (β = −0.056, *p* = 0.03) as well as with the social domain of QoL (β = 0.050, *p* = 0.035). The results show that a higher score in the physical domain was associated with lower HRBS; thus, H1 was supported. Unexpectedly, a higher score in the social domain was associated with higher HRBS; thus, H3 was not supported. In addition, the results show that the psychological and environmental domains were not significantly associated with HRBS. Thus, H2 and H4 were also not supported. Model 4 was used to analyze if the physical and social domains would still be significant indicators of HRBS after controlling for the socio-demographic variables. The results still held after controlling these confounding variables, suggesting that the physical domain of QoL (β = −0.056, *p* = 0.030) and social domain of QoL (β = 0.048, *p* = 0.041) were still significant contributors to HRBS. Moreover, gender (β = 0.122, *p* < 0.001), age (β = 0.048, *p* = 0.009), and education (β = −0.037, *p* = 0.042) were significant predictors of HRBS in Model 2, but residence and income were not statistically significant predictors. These results are consistent with the findings of the logistic regression analysis.

## 4. Discussion

Health rumors may mislead people to take adverse health treatment and engage in inappropriate disease-prevention behaviors. For health rumor governance, understanding the underlying mechanism of people’s belief in health rumors is of importance. Although previous studies have put forward insightful views on factors influencing HRB from different perspectives, whether a higher QoL serves as a protective factor in keeping people away from the negative effects of health rumors was still unknown. Thus, this study explored the relationship between individuals’ four domains of QoL and HRB by conducting a national cross-sectional survey study in China.

The first main finding was that the poorer an individual’s perception of their physical health or QoL, the higher the HRB, which was in accordance with the first hypothesis. This result is consistent with previous theoretical and empirical studies. According to the selective exposure theory [44], individuals would actively search for information closely related to themselves and selectively ignore irrelevant information. Therefore, people who are not satisfied with their health status will stimulate their subjective motivations by paying more attention to health-related information according to their condition, leading to wider contact with various health rumors, which further increases the probability to fall for them. Previous studies have also shown that cancer patients falsely believe rumors they think could make their situation better [45].

The result that people who reported higher satisfaction with the social domain of QoL were more likely to believe in health rumors is unexpected yet important. This interesting result is indirectly supported by some previous studies. For example, it has been argued that people with the “extraversion” personality trait are more vulnerable to rumors [19]. An “extraversion” personality is more likely to have social relationships with people, which may relate to higher satisfaction with their social QoL. For example, it has been found that the “extraversion” personality is positively associated with the social domain of QoL [46]. Moreover, since the credibility of the rumor source [47] and close social ties [23] are associated with rumor belief, individuals who are in a closer and harmonious interpersonal relationship may have a high degree of trust in each other, which increases people’s belief in health rumors circulating in interpersonal communication channels. In addition, some demographic variables were significantly associated with HRB. Specifically, the female, older adult, and the less educated groups were associated with a higher likelihood of rumor belief. These results are also consistent with prior research [19,48,49,50,51].

This research has practical implications for identifying people who are more vulnerable to health rumors and supporting intervention strategies to combat such rumors. The results show that an individual who is dissatisfied with their physical health is a robust predictor of HRB, which can provide a reference for identifying groups to protect against health rumors in the future. According to the information-seeking theory [52], those who are in a poor health state are more likely to seek out health-related information, which also makes them more likely to be exposed to health rumors. Therefore, medical personnel and institutions need to increase health science popularization and health rumor warning for such people. For example, when a patient visits a doctor, the doctor needs to warn the patient against misbelieving health rumors and inform them of the risks associated with such rumors. Besides, the findings also indicated that better-perceived satisfaction in social relationships of QoL is another predictor of HRB. Trust in interpersonal relationships, to a certain extent, endows health rumors with considerable credibility, causing challenges to the governance of such rumors. In turn, the interpersonal communication attribute of “strong ties” on online social platforms also provides new ideas for health rumor refuting. For example, the WeChat platform embedded a mini-program called “Piyao assistant” to popularize science to the public [53], through which users can share the correct information published by the program to closely connected friends and groups against rumors. Moreover, Facebook also takes advantage of the characteristics of interpersonal communication and adopts the means of marking false information in social networks [54]. Prior studies have concluded that sharing denials among the online friend community significantly helps spread the truth [55]. Future research should pay more attention to exploring which kinds of people are willing to share true information about health rumors on social media and designing strategies for stimulating users to participate in disseminating the truth.

Admittedly, there are some limitations to this study worth considering. First, the analytical data collected in this study come from a national cross-sectional survey; therefore, research results may present a robust correlation rather than causality. Future research should try to explore the causal relationship between various domains of QoL and HRB by employing the experiment method. The second is the application of the short version of the WHOQOL scale. Considering the length of the assessment, there is a risk that it may not cover an individual’s detailed assessment of life, which may have a certain degree of impact on research results. Future studies could adopt a more comprehensive measure of QoL to examine the connection between an individual’s QoL and health rumor belief. Finally, this study took place in China; given the differences of people in different cultural settings, future research could expand the conclusions of this study to other cultural backgrounds by exploring the relationship between QoL and HRB in different cultural backgrounds.

## 5. Conclusions

The purpose of this study was to explore the relationship between an individual’s QoL level and HRB. The results show that people with a poor perception of their physical health were more likely to believe in health rumors. Interestingly, an unexpected yet critical result was that individuals who had a better evaluation of their social relationships were more susceptible to health rumors. Moreover, the female group and older adults showed greater belief in health-related rumors. These results expand upon our understanding of people with different QoL perceptions interacting with false health rumors. The results also identify the characteristics of the rumor-susceptible population, providing several essential implications for online and offline interventions to govern health rumors in practice.

## Figures and Tables

**Table 1:** Sample characteristics (N = 3633).

Demographic Variables		N	%
Gender	Male	2077	55.0%
	Female	1556	45.0%
Age group	Below 18	283	7.4%
	18–29	1434	39.3%
	30–39	1250	34.5%
	40–49	580	16.2%
	50–69	86	2.4%
Residence	Urban area	2703	74.7%
	Rural area	930	25.3%
Education	Less than middle school	1193	32.8%
	Some college	840	23.1%
	Bachelor’s degree	1273	35.0%
	Master’s degree or higher	327	9.0%
Income	Below 2000	82	2.3%
	2001–6000	613	16.9%
	6001–10,000	817	22.5%
	10,001–15,000	795	21.9%
	15,000–30,000	694	19.1%
	30,001–45,000	255	7.0%
	45,000–60,000	96	2.6%
	Above 60,000	281	7.7%

**Table 2:** Scores of four major WHOQOL-BREF domains and HRB.

Statistics	WHOQOL-BREF Domains				HRB	
	Physical Domain	Psychological Domain	Social Domain	Environmental Domain	HRBS	FFHR
Minimum	4.0	4.0	4.0	4.0	1.0	0.0
Maximum	20.0	20.0	20.0	20.0	5.0	1.0
Mean	14.96	14.25	13.33	13.86	2.89	0.84
SD	2.74	3.07	3.37	3.09	0.84	0.37

HRBS—health rumor belief score, FFHR—falling for health rumor, SD—standard deviation.

**Table 3:** Binary logistic regression analysis for predicting FFHR by WHOQOL-BREF domains.

Independent Variables	Model 1					Model 2				
	B	SE	OR	95% CI	*p*-Value	B	SE	OR	95% CI	*p*-Value
Physical domain	−0.058 *	0.029	0.944	0.892–0.998	0.043	−0.060 *	0.029	0.942	0.890–0.996	0.037
Psychological domain	−0.014	0.027	0.986	0.935–1.040	0.602	−0.018	0.027	0.982	0.930–1.036	0.506
Social domain	0.036 ^+^	0.021	1.037	0.995–1.080	0.083	0.036 ^+^	0.021	1.036	0.995–1.080	0.088
Environmental domain	−0.013	0.025	0.988	0.940–1.038	0.622	−0.019	0.026	0.981	0.931–1.033	0.465
Gender						0.477 **	0.106	1.611	1.310–1.982	0.000
Age						0.201 **	0.060	1.222	1.087–1.374	0.001
Residence						0.247	0.132	1.280	0.988–1.657	0.061
Education						−0.022	0.054	0.979	0.880–1.088	0.689
Income						−0.006	0.030	0.994	0.936–1.055	0.843

* *p* < 0.05, ** *p* < 0.01, ^+^ *p*-marginal significant. FFHR—falling for health rumor, SE—standard error, OR—odds ratio, CI—confidence interval.

**Table 4:** Linear regression analysis for predicting HRBS by WHOQOL-BREF domains.

Independent Variables	Model 3					Model 4				
	β	SE	B	95% CI	*p*-Value	β	SE	B	95% CI	*p*-Value
Physical domain	−0.056	0.008	−0.018	−0.035–−0.002	0.030	−0.056	0.008	−0.018	−0.034–−0.002	0.030
Psychological domain	−0.003	0.008	−0.001	−0.017–0.015	0.908	−0.002	0.008	−0.001	−0.016–0.015	0.944
Social domain	0.050	0.006	0.013	0.001–0.025	0.035	0.048	0.006	0.013	0.000–0.025	0.041
Environmental domain	0.002	0.008	0.000	−0.014–0.015	0.948	−0.012	0.008	−0.003	−0.019–0.012	0.653
Gender						0.122	0.030	0.219	0.161–0.277	0.000
Age						0.048	0.017	0.046	0.012–0.080	0.009
Residence						−0.009	0.038	−0.019	−0.093–0.056	0.622
Education						−0.037	0.016	−0.032	−0.064–−0.001	0.042
Income						−0.009	0.009	−0.005	−0.022–0.013	0.605
Adjusted R^2^	0.001					0.020				
*F*	*F*(4, 3628) = 2.112					*F*(9, 3623) = 9.201 **				

** *p* < 0.01. HRBS—health rumor belief score, β—standardized coefficients, SE—standard error, B—unstandardized coefficients, CI—confidence interval.

## Data Availability

The data presented in this study are available on request from the corresponding author.
